# Changing Prescription Patterns in Pharmacologically Treated Patients with Newly Diagnosed Inflammatory Bowel Disease in German Gastroenterology Practices: A Real-World Study, 2015–2024

**DOI:** 10.3390/ph19101590

**Published:** 2026-10-08

**Authors:** Annika Fernandez Milano, Sarah Krieg, Karel Kostev

**Affiliations:** 1 Epidemiology, IQVIA, 60549 Frankfurt am Main, Germany; 2 Department of Inclusive Medicine, University Clinic for People with Neurodevelopment Disorders, Mara Hospital, Medical School and University Medical Center OWL, Bielefeld University, 33617 Bielefeld, Germany; 3 Philipps-University, University Clinic, 35043 Marburg, Germany

**Keywords:** inflammatory bowel disease, Crohn’s disease, ulcerative colitis, prescription patterns, biologics, real-world data, Germany

## Abstract

**Background/Objectives:** The therapeutic landscape of inflammatory bowel disease (IBD) has evolved, particularly with the expanding availability of biologic and targeted therapies. This study characterized real-world prescribing patterns among patients with newly diagnosed Crohn’s disease (CD) and ulcerative colitis (UC) in Germany. **Methods:** This retrospective study used the German IQVIA Disease Analyzer database. Patients aged 15–80 years diagnosed with CD or UC between 2015 and 2024, with ≥12 months of follow-up and at least one predefined treatment prescription within 12 months after diagnosis, were included. Prescription patterns were compared by IBD subtype, diagnosis period (2015–2019 vs. 2020–2024), age, and sex. Associations were assessed using multivariable logistic regression. **Results:** Overall, 10,009 patients were included (CD, n = 3810; UC, n = 6199). Mesalazine remained predominant in UC (89.7% in 2015–2019 vs. 87.9% in 2020–2024), whereas biologics were prescribed more frequently in CD and were more common in the later diagnosis period (CD: 32.2% vs. 54.0%; UC: 14.6% vs. 21.5%). UC was associated with higher odds of mesalazine prescription (OR 15.17) and lower odds of biologic prescription (OR 0.28) compared with CD. The later diagnosis period was associated with higher odds of biologic prescription (OR 2.00) and lower odds of mesalazine (OR 0.67) and thiopurine prescription (OR 0.59). Prescription patterns also differed by age, whereas sex-related differences were limited. **Conclusions:** Among pharmacologically treated patients with newly diagnosed IBD, prescription patterns differed substantially between diagnosis periods, with more frequent biologic and less frequent thiopurine and mesalazine prescriptions in the later period, particularly in CD. Patterns also differed by IBD subtype and age.

## 1. Introduction

Crohn’s disease (CD) and ulcerative colitis (UC), the two major forms of inflammatory bowel disease (IBD), are chronic immune-mediated inflammatory disorders of the gastrointestinal tract [1]. Their prevalence has increased substantially worldwide, resulting in a considerable and growing disease burden [2]. In Germany, it is estimated that there are approximately 250,000 patients with CD and 400,000 with UC [3]. UC is characterized by continuous mucosal inflammation of the colon, typically beginning in the rectum and extending proximally to a variable extent, whereas CD is characterized by discontinuous, transmural inflammation that may affect any part of the gastrointestinal tract, most commonly the terminal ileum and colon [1].

Current IBD therapies primarily target immune dysregulation and intestinal inflammation [4], and aim to achieve corticosteroid-free clinical remission and endoscopic healing, normalize inflammatory biomarkers, restore quality of life, and prevent disease progression and complications. Histological healing in UC and transmural healing in CD may provide additional information on the depth of remission but are not currently considered formal treatment targets [5,6,7]. In addition to pharmacological therapy, nutritional interventions and the optimization of nutritional status represent important components of comprehensive IBD care [3,8], while surgery remains an integral treatment option for selected patients [3].

In Germany, treatment decisions are primarily guided by the S3 guidelines for UC and CD [6,9], which are largely consistent with European Crohn’s and Colitis Organisation (ECCO) recommendations [10,11]. Therapeutic strategies are based on disease activity, phenotype, prognostic risk factors, previous treatment response, comorbidities, safety considerations, and patient preferences [10,11]. For mild-to-moderate UC, mesalazine (5-aminosalicylic acid, 5-ASA) represents the standard induction therapy, with locally acting or systemic corticosteroids used in cases of inadequate response or higher disease activity [6]. In contrast, budesonide is recommended for induction in selected patients with mild-to-moderate CD limited to the ileum and/or right colon, while systemic corticosteroids may be used in more extensive or severe disease. 5-ASA has no established role in contemporary medical management of CD and is not recommended by the current S3 guideline for either induction or maintenance therapy [9].

Patients with moderate-to-severe disease, corticosteroid dependence or refractoriness, inadequate response to conventional treatment, or other high-risk features may require escalation to steroid-sparing advanced therapies. These include biologics targeting tumor necrosis factor (TNF), integrins, interleukin (IL)-12/23, and IL-23 pathways, as well as targeted synthetic therapies such as Janus kinase (JAK) inhibitors and, in UC, sphingosine-1-phosphate (S1P) receptor modulators [6,9]. The number and mechanistic diversity of available advanced therapies expanded substantially over the past decade, enabling increasingly individualized treatment strategies [12]. In selected patients, combination therapy with an anti-TNF agent and a thiopurine may be considered, while calcineurin inhibitors have a specific role as rescue therapy in acute severe UC. Following successful induction, effective steroid-sparing treatment should generally be continued as maintenance therapy, whereas corticosteroids should not be used for maintenance because of their unfavourable long-term efficacy and safety profile [6,9].

These differences in treatment recommendations may translate into distinct real-world prescribing patterns between UC and CD. Changes in available treatment options may also be reflected in prescribing patterns, particularly in the period following diagnosis. Although several studies have investigated real-world treatment patterns in IBD, including treatment during the first year following diagnosis [13,14,15,16,17,18], evidence on contemporary pharmacological management of newly diagnosed patients in office-based gastroenterology practices in Germany remains limited. A nationwide German study by Weylandt et al. examined annual prescription patterns among prevalent patients with CD and UC between 2012 and 2020 but did not specifically assess treatment patterns following diagnosis [19]. Two further German studies have examined treatment sequences and outcomes among patients receiving advanced therapies [20,21] but provide limited insight into the broader spectrum of pharmacological treatment following IBD diagnosis. Consequently, previous evidence may not fully reflect contemporary prescribing patterns among newly diagnosed patients in Germany. Therefore, this study aimed to characterize real-world prescription patterns in the period around an initial diagnosis of CD or UC in office-based gastroenterology practices in Germany and to examine differences according to IBD subtype, diagnosis period, age, and sex.

## 2. Results

### 2.1. Baseline Characteristics of the Study Population

The 6199 UC patients were on average older than the 3810 CD patients (mean age 44.3 [standard deviation (SD) 15.8] vs. 40.5 [15.5] years; *p* < 0.001) and were more frequently older than 45 years (45.9% vs. 37.9%), whereas CD patients were more often aged 15–30 years (32.7% vs. 23.3%) (Table 1). Women accounted for 54.8% of CD but 49.6% of UC patients (*p* < 0.001). The distribution of the diagnosis periods was comparable between the two groups (diagnosis in 2020–2024: 58.5% of CD and 57.1% of UC patients; *p* = 0.148). CD patients had a slightly higher median number of physician visits per year during follow-up than UC patients (8 [interquartile range (IQR) 5–11] vs. 6 [IQR 3–10]; *p* < 0.001).

### 2.2. Prescription Patterns by Diagnosis Period

**Figure 1:**
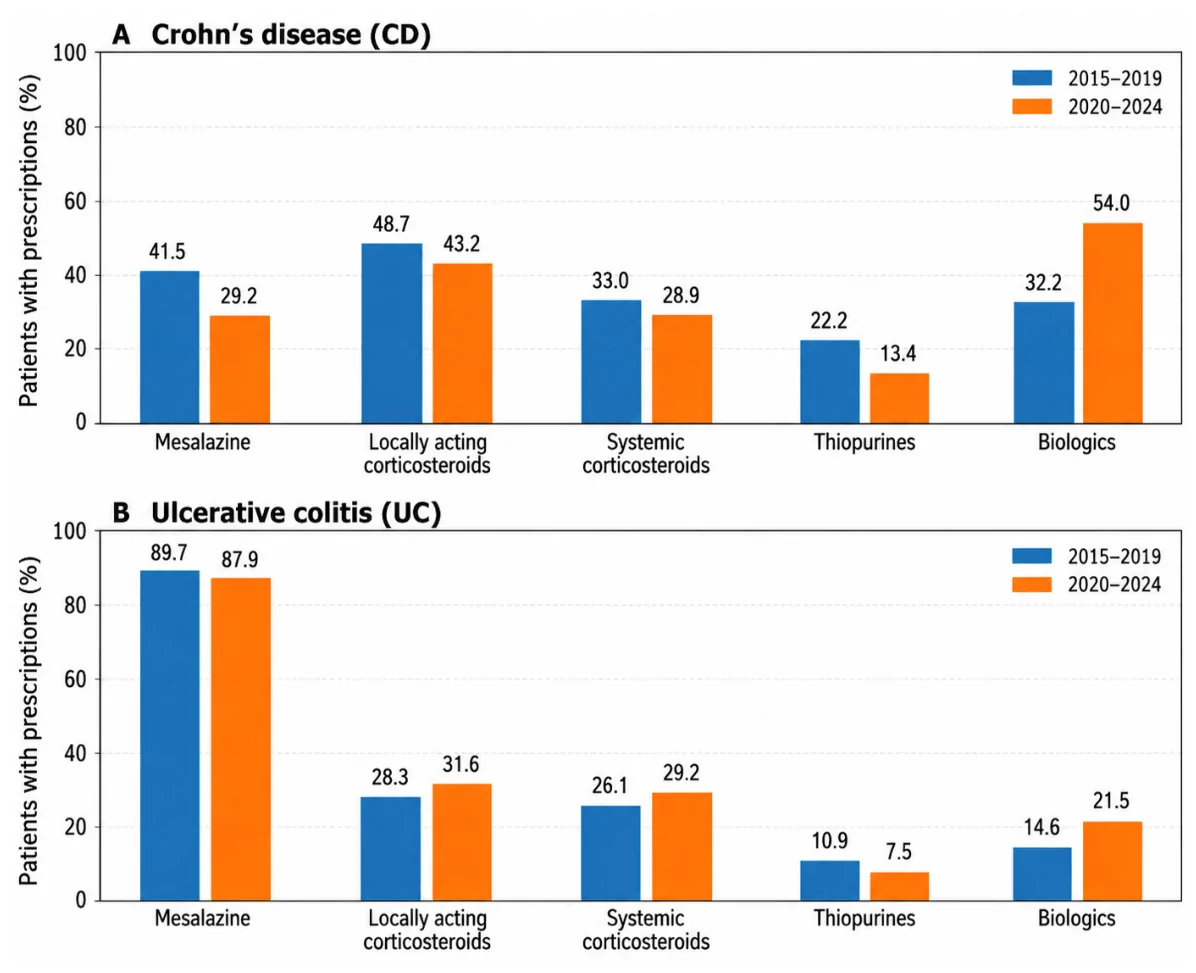
Prescription patterns among patients with CD (**A**) and UC (**B**) with at least one recorded prescription for a predefined treatment class during the observation period, stratified by diagnosis period (2015–2019 and 2020–2024). Methotrexate, calcineurin inhibitors, and targeted synthetic therapies were omitted from the figure due to the small proportions of patients with prescriptions for these treatment classes.

Prescription patterns differed substantially between CD and UC and between the two diagnosis periods (Table 2, Figure 1). In both periods, mesalazine was by far the most frequently prescribed therapy in UC patients (89.7% and 87.9% in 2015–2019 and 2020–2024, respectively), compared with 41.5% and 29.2% in CD patients, respectively (both *p* < 0.001). Locally acting corticosteroids, thiopurines, and biologics were consistently prescribed more often in CD than in UC patients. Biologic prescriptions were more frequent in the later diagnosis period, particularly in CD patients (54.0% vs. 32.2%), while remaining lower in UC patients (21.5% vs. 14.6%). Conversely, thiopurine prescriptions were less frequent in the later diagnosis period, particularly in CD patients. Targeted synthetic therapies remained rare but were prescribed more frequently in UC than in CD patients (up to 2.2%). Differences between CD and UC were statistically significant (*p* < 0.001) for most treatment classes, except for systemic corticosteroids in the 2020–2024 diagnosis period, and methotrexate and calcineurin inhibitors in both periods.

### 2.3. Prescription Patterns by Sex

Stratification by sex revealed largely comparable prescription patterns among women and men (Table 3). Mesalazine was prescribed to approximately 88–89% of patients with UC and 33–36% of those with CD, irrespective of sex. Conversely, biologics were prescribed more frequently in CD (45%) than in UC (17–20%) in both women and men. Systemic corticosteroids and thiopurines were prescribed slightly more often in men than in women, whereas locally acting corticosteroids were prescribed somewhat more frequently in women. Differences between CD and UC remained statistically significant (*p* < 0.001) for mesalazine, locally acting corticosteroids, thiopurines, biologics and targeted synthetic therapies, but not for systemic corticosteroids, methotrexate, or calcineurin inhibitors.

### 2.4. Prescription Patterns by Age Group

Stratification by age group (Table 4) showed age-related differences in prescribing patterns. Biologic prescriptions were highest in the youngest CD patients (56.3% in those aged 15–30 years) and declined with age (43.0% and 36.7% in those aged 31–45 and >45 years, respectively). A similar trend was observed in UC patients, but at a substantially lower level than in CD patients (26.2%, 17.9%, and 15.1%, respectively). Mesalazine was prescribed to 88–90% of UC patients regardless of age, whereas its prescriptions in CD increased with age (29.9%, 33.7%, and 38.7%, respectively). Thiopurines were prescribed more frequently in CD than UC across all age groups but less often in patients aged >45 years. Systemic corticosteroid prescriptions, however, declined with age in both diseases. Differences between CD and UC were significant (*p* < 0.001) for mesalazine, locally acting corticosteroids, thiopurines, biologics, and targeted synthetic therapies in all age groups, whereas systemic corticosteroids, methotrexate, and calcineurin inhibitors did not differ significantly.

### 2.5. Factors Associated with Treatment Prescriptions

In the multivariable logistic regression models (Table 5), UC compared with CD was strongly and positively associated with the prescription of mesalazine (odds ratio [OR] 15.17; 95% confidence interval [CI] 13.66–16.84) and targeted synthetic therapies (OR 18.14; 95% CI 6.68–49.25), and inversely associated with biologics (OR 0.28; 95% CI 0.26–0.31), thiopurines (OR 0.49; 95% CI 0.43–0.55) and locally acting corticosteroids (OR 0.53; 95% CI 0.48–0.57) (all *p* < 0.001). Compared with the 2015–2019 diagnosis period, diagnosis in 2020–2024 was associated with lower odds of mesalazine (OR 0.67) and thiopurine prescriptions (OR 0.59) and higher odds of biologic prescriptions (OR 2.00) (all *p* < 0.001). Male vs. female sex was associated with higher odds of systemic corticosteroid (OR 1.17) and thiopurine prescriptions (OR 1.25), and lower odds of locally acting corticosteroid prescriptions (OR 0.85). Relative to patients aged 15–30 years, patients aged 31–45 and >45 years had lower odds of systemic corticosteroid and biologic prescriptions and, among those aged >45 years, thiopurine prescriptions (all *p* < 0.001).

### 2.6. Treatment Sequences and Combinations

The first prescribed treatment class differed between CD and UC. In CD, locally acting corticosteroids (28.2%) and biologics (24.0%) were most frequently prescribed first, whereas mesalazine was the first prescribed treatment in 63.1% of UC patients (Table S1). Among CD patients with locally acting corticosteroid prescriptions, 38.3% did not receive any main treatment during the observation period, 27.8% received locally acting corticosteroids before, 14.5% on the same day as, and 19.4% after the first main treatment. TNF inhibitors and thiopurines were co-prescribed in 11.7% of CD and 13.8% of UC patients receiving TNF inhibitors. Mesalazine was co-prescribed in 14.1% of CD and 61.1% of UC patients receiving biologics, and systemic corticosteroids were co-prescribed in 29.2% of CD and 45.1% of UC patients receiving vedolizumab (Table S2). Among 1148 UC patients with a biologic prescription, 52.3% had a mesalazine prescription before or on the date of the first biologic prescription, and 49.0% received mesalazine after biologic initiation. Overall, 33.1% had mesalazine prescriptions both before and after biologic initiation, corresponding to 63.3% of those previously treated with mesalazine (Table S2).

## 3. Discussion

In this large real-world study of pharmacologically treated patients with newly diagnosed IBD managed in office-based gastroenterology practices in Germany, several important findings emerged. First, prescription patterns differed substantially between patients diagnosed in 2015–2019 and 2020–2024, with prescriptions increasing for biologics and decreasing for thiopurines and mesalazine in the later diagnosis period, particularly among patients with CD. Second, prescription patterns differed markedly between CD and UC, with mesalazine being the predominant therapy in UC, whereas biologics, thiopurines, and locally acting corticosteroids were prescribed more frequently in CD. Third, prescription patterns differed across age groups, whereas they were largely comparable between women and men. These associations persisted in the multivariable analyses.

Prescription patterns differed considerably between the two diagnosis periods. In the later period, biologic prescriptions were substantially more frequent in CD (54.0% vs. 32.2%) and, to a lesser extent, in UC (21.5% vs. 14.6%). In contrast, thiopurine and mesalazine prescriptions were less frequent in the later period, particularly among patients with CD. In the multivariable analyses, diagnosis period remained independently associated with the observed prescription patterns. Compared with the ECCO Epidemiological Committee (EpiCom) inception cohort of patients newly diagnosed with IBD in Western Europe in 2010, our findings are consistent with a continued shift toward greater use of biologics and lower use of immunomodulators and mesalazine [18]. Similar developments have been reported in studies from Germany, Spain, and China [13,19,22], although differences in study populations, treatment definitions, observation periods, and reporting metrics limit direct comparisons.

In the German study by Weylandt et al., biologic use increased, and thiopurine use decreased between 2012 and 2020, while mesalazine use declined in CD and remained relatively stable in UC [19]. Similarly, the Catalan population-based registry study by Brunet-Mas et al. demonstrated increasing biologic use, particularly in CD patients (25.1% to 38.2%), alongside decreasing use of conventional immunosuppressants between 2017 and 2023 [22]. Although the reported treatment proportions in the Catalan study were lower because untreated patients were included and treatment classifications differed, these findings are consistent with the observed shift toward biologic therapies. Comparable developments were reported by Yao et al. in China between 1999 and 2020, including declining 5-ASA use among patients with CD and increasing infliximab use in both CD and UC [13].

These temporal changes may reflect several developments in IBD management, including evolving ECCO and German S3 guideline recommendations and the increasing emphasis on early effective and steroid-sparing treatment. At the same time, treatment selection has become increasingly individualized based on disease- and patient-related factors [6,9,10,11]. In addition, the introduction and increasing uptake of biosimilars may have facilitated broader access to biologic therapies by reducing treatment costs [23]. Furthermore, concerns regarding the long-term safety of thiopurines, including increased risks of lymphoma, non-melanoma skin cancer, and infections [24], may have contributed to their declining use. Finally, the increasing availability of biologic and targeted synthetic therapies, including anti-integrin, anti-IL-12/23, anti-IL-23, JAK inhibitors, and S1P modulators for UC [6,9], has expanded the therapeutic armamentarium in recent years. Although the two diagnosis periods were defined as periods of equal length, the second period coincides with the approval of several new advanced therapies for IBD, including filgotinib, ozanimod, upadacitinib, risankizumab, mirikizumab, and etrasimod, as well as subcutaneous formulations of infliximab and vedolizumab, which may have facilitated the use of these agents in office-based practices [6,9,12]. In addition, therapies approved towards the end of the first period, such as tofacitinib and ustekinumab for UC, as well as adalimumab biosimilars, are likely to have been adopted mainly during the second period.

Prescription patterns also differed substantially between CD and UC, consistent with disease-specific treatment recommendations. Mesalazine remained by far the most frequently prescribed therapy in UC, being prescribed to more than 85% of patients in both diagnosis periods. In contrast, mesalazine was prescribed considerably less frequently in CD, with prescriptions declining from 41.5% to 29.2% in the later diagnosis period. Similar differences have been reported in other cohorts. In the Inflammatory Bowel Disease in South-Eastern Norway III (IBSEN III) study of incident IBD patients diagnosed between 2017 and 2019, mesalazine use was common in UC (81.3%) but substantially less frequent in CD (17.8%) [16,17]. Likewise, a Spanish study of IBD patients diagnosed in 2017 reported higher mesalazine use in UC (73%) than in CD (38%), although direct comparisons are limited by differences in study design and the inclusion of untreated patients [25]. The differences are consistent with the established role of 5-ASA in mild-to-moderate UC, whereas its use is not recommended in current CD guidelines [6,9,10,11]. Nevertheless, the continued prescription of mesalazine in almost one-third of patients diagnosed with CD in 2020–2024 is noteworthy. Several explanations are possible. First, mesalazine has historically been used in patients with mild colonic CD, and some clinicians may still consider it an option in this setting despite limited evidence of efficacy [26]. Second, mesalazine is well tolerated and inexpensive, which may favor its use in patients with mild symptoms or in those reluctant to receive immunosuppressive therapy. The decrease from 41.5% to 29.2% between the two diagnosis periods suggests an increasing implementation of current guideline recommendations. However, as information on disease location, disease activity, and reasons for treatment selection was not available in the database, these explanations remain speculative.

In contrast, biologics and thiopurines were prescribed more frequently in CD than in UC. Similar patterns were observed in both the IBSEN III and Spanish cohorts, which reported higher use of immunomodulators and biologics among CD than among UC patients [16,17,25]. The greater use of biologics in CD may partly reflect differences in disease biology and treatment requirements. CD is characterized by transmural inflammation and may result in cumulative bowel damage, including stricturing and penetrating complications [27]. Increasing evidence supports early effective therapy in patients with active CD, particularly in those at risk of disease progression. Recent randomized data from the PROFILE trial demonstrated substantially higher rates of sustained steroid- and surgery-free remission with early top-down infliximab-based combination therapy than with an accelerated step-up strategy in newly diagnosed active CD [28]. By comparison, many patients with mild-to-moderate UC can achieve disease control with conventional first-line therapy such as mesalazine [6,11,27]. However, differences in disease activity, phenotype, and prognostic factors, which strongly influence treatment selection in both CD and UC [10,11], were unavailable in our database and may have contributed to the observed prescription patterns.

Locally acting corticosteroids were also more frequently prescribed in CD, potentially reflecting differences in disease location and the established role of oral budesonide for induction of remission in patients with mild-to-moderate ileocecal and/or right-sided colonic disease [9]. In contrast, systemic corticosteroid prescription patterns were relatively similar between CD and UC, with around one quarter to one third of patients receiving systemic corticosteroid prescriptions during the observation period. This aligns with what was reported in the IBSEN III study [16,17]. Although corticosteroids remain an important option for induction of remission, their frequent prescription around the time of diagnosis is noteworthy in the context of current treatment goals emphasizing corticosteroid-free disease control [6,9]. Notably, the proportion of patients with systemic corticosteroid prescriptions did not decrease between the two diagnosis periods despite the increasing use of biologics. Corticosteroids remain the standard option for rapid induction of remission in moderate-to-severe active disease and are frequently used as bridging therapy until slower-acting steroid-sparing agents, such as thiopurines or vedolizumab, become effective [6,9]. As the first year after diagnosis is the period in which most patients require induction therapy, increasing use of advanced therapies may not necessarily be accompanied by fewer corticosteroid prescriptions during this period. However, as our data do not allow differentiation between short induction courses and prolonged, repeated, or steroid-dependent use, it remains unclear to what extent the unchanged proportion reflects appropriate short-term use rather than avoidable corticosteroid exposure. Methotrexate, calcineurin inhibitors, and targeted synthetic therapies were rarely prescribed during the study period, which may reflect their more specific therapeutic roles and, in the case of targeted synthetic therapies, their relatively recent availability [3,6,9].

Age was significantly associated with prescription patterns, with younger patients more frequently prescribed biologics, thiopurines, and systemic corticosteroids. Similar age-related differences have been reported previously. Nørgård et al. found that elderly-onset IBD patients were less likely to initiate biologics or thiopurines within the first year after diagnosis [29], while Everhov et al. similarly reported lower use of biologics and immunomodulators among elderly-onset than adult-onset IBD patients [30]. These findings are further supported by an expert review by Ananthakrishnan et al. [31]. Regarding corticosteroids, Nørgård et al. found that elderly patients were less likely to initiate but more likely to continue treatment than adults [29]. Everhov et al. reported contrasting first-year patterns for CD and UC, followed by higher use among elderly patients in both groups thereafter [30]. Differences in age categorization limit direct comparisons, as previous studies compared adult-onset with elderly-onset IBD, usually defined as onset at 60 years or older [29,30,31], whereas our oldest age group (>45 years) also included middle-aged patients and was not intended to represent elderly-onset IBD. Nevertheless, the findings are consistent with age-related differences in biologic and immunomodulator prescriptions. A greater burden of comorbidities and polypharmacy, and increased vulnerability to treatment-related complications, including serious infections and malignancies, may lead to more cautious use of immunomodulators and biologics with increasing age. At the same time, greater reliance on corticosteroids has been reported in older IBD populations, potentially reflecting concerns about adverse events or reduced efficacy of steroid-sparing therapies [24,32]. In contrast, systemic corticosteroid prescriptions were less frequent with increasing age in our study, possibly related to greater caution regarding their adverse effects [24]. However, unmeasured differences in clinical characteristics between age groups may also have contributed.

Prescription patterns were broadly similar between sexes, with higher odds of thiopurine and systemic corticosteroid prescriptions among men and lower odds of locally acting corticosteroid prescriptions compared with women in the multivariable models (*p* < 0.001). Similar differences have been reported by Heath et al., who observed greater immunomodulator and prednisone use among men and greater budesonide use among women [33]. Overall, sex-related differences in IBD treatment have been inconsistent, with a recent systematic review reporting no significant sex differences in most treatment comparisons [34]. Some treatment options, including methotrexate, JAK inhibitors, and S1P receptor modulators, are contraindicated during pregnancy, which may limit their use in women of reproductive age [35]. In our study, targeted synthetic therapies were more frequently prescribed in men than in women (OR 1.52; 95% CI 1.05–2.22), although this association did not reach the predefined level of statistical significance. Because methotrexate and targeted synthetic therapies were rarely prescribed, a separate analysis of women of reproductive age was not feasible.

The additional analyses provide further insight into treatment sequences and combinations. In CD, locally acting corticosteroids were mostly prescribed as monotherapy or before other treatments, consistent with the role of budesonide as first-line induction therapy in mild-to-moderate ileocecal disease [9]. Combination therapy with TNF inhibitors and thiopurines, which has been shown to be more effective than monotherapy with either agent in CD [36], was prescribed infrequently, possibly reflecting concerns regarding the long-term safety of thiopurines [24]. In UC, mesalazine was continued after biologic initiation in almost two-thirds of previously treated patients, although discontinuation of 5-ASA in patients starting biologic therapy has not been associated with adverse clinical outcomes [37]. The frequent co-prescription of corticosteroids with vedolizumab is consistent with their use as bridging therapy until the onset of action of this slower-acting agent. These findings should be interpreted with caution, as co-prescription within a 90-day window does not necessarily indicate concomitant use, and intravenously administered biologics may be incompletely captured in office-based practice data.

A major strength of this study is the large number of patients managed in routine care in office-based gastroenterology practices, which enhances the generalizability of the findings to patients with IBD treated in office-based gastroenterology practices in Germany. Furthermore, the restriction to patients with a first recorded IBD diagnosis and the assessment of prescriptions in the period around diagnosis provide insight into early prescription patterns in clinical practice. In addition, the 10-year study period enabled comparison of prescription patterns amid substantial changes in IBD treatment. The inclusion of both CD and UC within the same population and healthcare setting allowed direct comparison of disease-specific prescription patterns under consistent methodological conditions. The combination of descriptive and multivariable analyses allowed us to examine associations between patient characteristics, IBD subtype, and diagnosis period with prescriptions of individual treatment classes. Finally, the broad range of conventional and advanced therapies captured in the study provides a comprehensive overview of prescription patterns in this setting.

Several limitations should be acknowledged. First, as the Disease Analyzer database is based on routinely collected practice data, information on important clinical factors such as disease activity, severity, phenotype, prognostic risk factors, treatment goals, and reasons for treatment selection was unavailable. These factors may substantially influence treatment choice, and residual confounding therefore cannot be excluded. Accordingly, the observed associations should not be interpreted as causal effects or as evidence of treatment appropriateness. Second, the database captures patients and treatments recorded in participating office-based gastroenterology practices; care provided in other healthcare settings was not captured, potentially leading to an underestimation of prescriptions, particularly for therapies administered in specialized or hospital-based settings. As the study population was restricted to patients managed in office-based gastroenterology practices, patients with more severe disease, particularly those requiring hospitalization at diagnosis or treated primarily in hospital-based or tertiary care centers, are likely underrepresented. Hospital admissions are not captured in the database; therefore, patients who required hospitalization at diagnosis could neither be identified nor excluded, and for patients initially diagnosed in hospital, the index date corresponds to the first documentation of IBD in the practice. Furthermore, only mesalazine (Anatomical Therapeutic Chemical [ATC] code A07EC02) was included, whereas other 5-ASA agents were not considered, potentially underestimating overall 5-ASA prescriptions. Also, recorded prescriptions do not necessarily reflect actual medication intake or adherence. Third, newly diagnosed IBD was defined based on the first recorded CD or UC diagnosis in the participating practice. Without a sufficiently long diagnosis-free look-back period, some patients with pre-existing IBD may have been classified as newly diagnosed, while reliance on routinely recorded International Classification of Diseases, 10th revision (ICD-10) codes may have introduced diagnostic misclassification. Excluding patients with both CD and UC codes during follow-up may also have resulted in the omission of patients undergoing diagnostic reclassification early in the disease course. Fourth, the analysis was restricted to patients with at least one recorded prescription for a predefined treatment class during the observation period, excluding 28.3% of otherwise eligible patients. The reported treatment proportions therefore apply to the treated analytic population and should not be generalized to all patients with newly diagnosed IBD. Fifth, the analysis assessed whether individual treatment classes were prescribed during the observation period but not their duration, cumulative exposure, or repeated courses, and treatment sequences and combinations could only be assessed descriptively based on prescription dates; clinical response was also unavailable. Consequently, the observed prescription patterns cannot be interpreted as measures of treatment effectiveness or detailed treatment trajectories. This is particularly relevant for corticosteroids, as the data do not allow differentiation between a single induction course and prolonged, repeated, or steroid-dependent treatment. Because very few patients received methotrexate or calcineurin inhibitors, the corresponding regression estimates were imprecise and should be interpreted with caution. Finally, temporal comparisons should be interpreted in the context of substantial changes in IBD treatment availability and clinical practice during the 10-year study period. Changes in coding, prescribing practices, and data completeness may also have introduced measurement variability, while the COVID-19 pandemic may have affected healthcare utilization and prescription patterns during 2020–2022.

## 4. Materials and Methods

### 4.1. Data Source

This retrospective study was based on data from the IQVIA Disease Analyzer (DA) database, which contains anonymized longitudinal electronic medical records from participating office-based physicians in Germany. Diagnoses are coded according to the International Classification of Diseases, 10th revision (ICD-10), and prescriptions according to the Anatomical Therapeutic Chemical (ATC) classification system of the World Health Organization (WHO). The database also includes basic demographic information. The sampling method of the DA database is based on statistics from the German Medical Association and accounts for physician specialty, German federal state, community size category, and physician age. A previous validation study showed that the overall panel of practices included in the DA database is broadly representative of office-based medical care in Germany [38]. The database has also been used in previous studies on IBD [39,40]. In Germany, ambulatory care, including specialist care, is predominantly provided by office-based physicians, whereas hospital outpatient clinics are involved in outpatient care only to a limited extent. The present study included only prescriptions issued in the participating office-based gastroenterology practices; prescriptions issued by general practitioners, in hospital outpatient clinics, or during inpatient stays were not captured. Information on a possible affiliation of the practices with hospitals was not available.

### 4.2. Study Population

**Figure 2:**
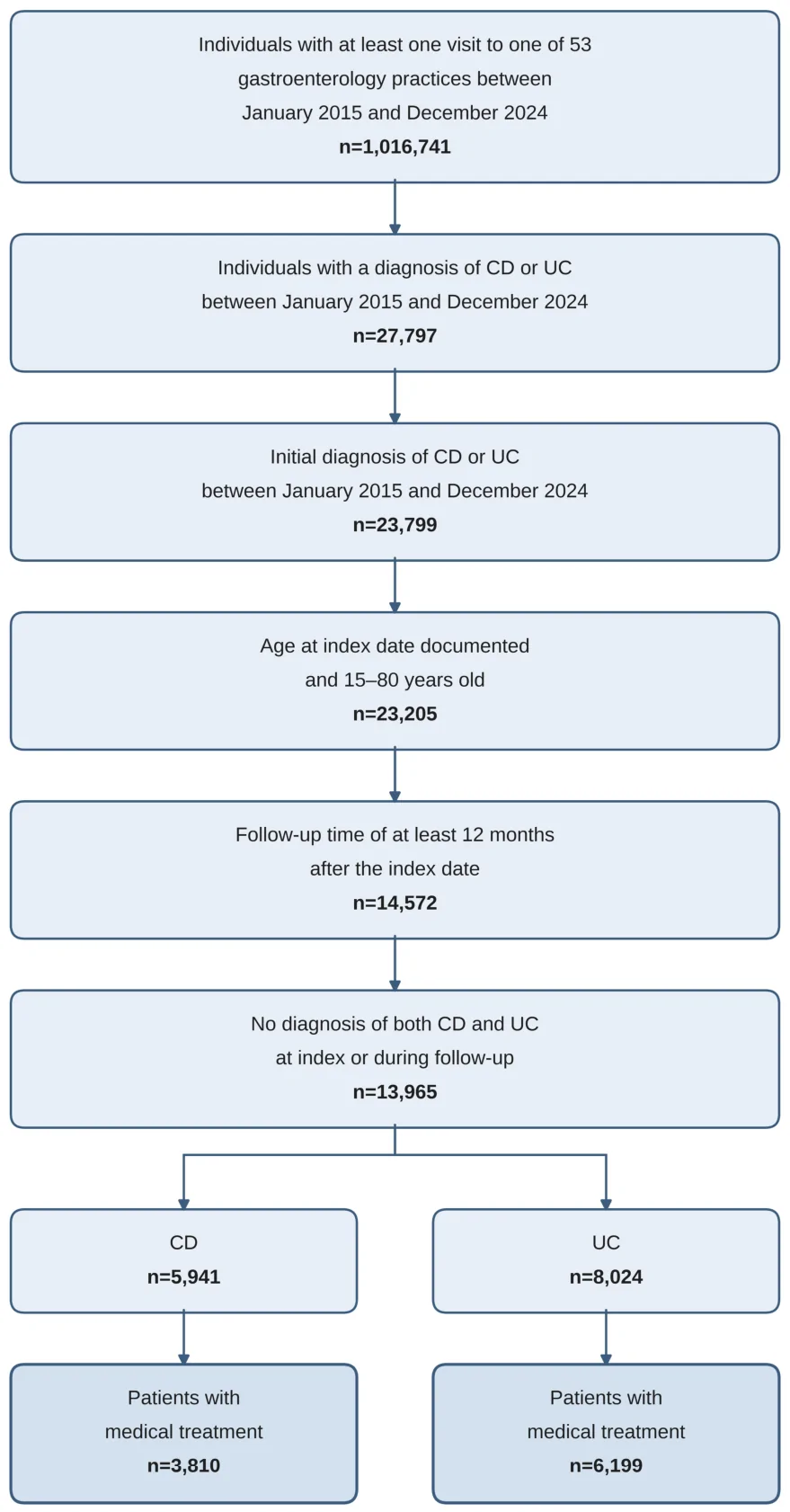
Selection of study population. CD, Crohn’s disease; UC, ulcerative colitis.

This study included all patients with a first recorded diagnosis (index date) of CD (ICD-10 “K50”) or UC (ICD-10 “K51”) documented in one of 53 gastroenterology practices in Germany between January 2015 and December 2024 (Figure 2). Of 1,016,741 individuals with at least one visit to these practices during the study period, 27,797 had a documented diagnosis of CD or UC, of whom 23,799 had their first recorded diagnosis within the study period. Further inclusion criteria were an age of 15–80 years at the index date (n = 23,205) and availability of at least 12 months of follow-up after the index date (n = 14,572). Patients with diagnoses of both CD and UC at the index date or during follow-up were excluded, leaving 13,965 patients (5941 with CD and 8024 with UC). The analysis was restricted to patients with at least one recorded prescription for one of the predefined pharmacological treatment classes within 12 months after the index date (observation period), resulting in a final analytic cohort of 3810 patients with CD and 6199 with UC. For patients diagnosed in 2024, the observation period could therefore extend into 2025. Of the 13,965 otherwise eligible patients, 3956 (28.3%) had no recorded prescription for any of the predefined treatment classes during the observation period and were therefore excluded from the analysis.

For each patient, all prescriptions within the observation period, i.e., within 12 months after the index date, were extracted and grouped into eight predefined treatment classes: 5-ASA, locally acting corticosteroids, systemic corticosteroids, thiopurines, methotrexate, calcineurin inhibitors, biologics, and targeted synthetic therapies. The active ingredients and the corresponding ATC codes of each treatment class are shown in Table 6. Treatment classes were not mutually exclusive; thus, patients could be prescribed more than one class during the observation period.

### 4.3. Statistical Analyses

Baseline characteristics of patients with CD and UC were compared using the Wilcoxon rank-sum test for continuous variables and the chi-square test for categorical variables. The proportion of patients with at least one prescription for each predefined treatment class during the observation period was compared between CD and UC overall and within strata defined by the diagnosis period (2015–2019 and 2020–2024), sex (male and female), and age group (15–30, 31–45, and >45 years) using chi-square tests. The two diagnosis periods were defined as periods of equal length (five years each). Age groups were defined based on the frequency distribution of age at diagnosis to obtain groups of sufficient size for stratified analyses.

For each treatment class, a multivariable logistic regression model was fitted, with at least one prescription of the respective treatment class during the observation period as the dependent variable. IBD subtype (UC vs. CD), diagnosis period (2020–2024 vs. 2015–2019), sex (male vs. female), age group (15–30 years as reference), and insurance status (private vs. statutory) were included simultaneously as independent variables; thus, the association of each variable was adjusted for the other covariates in the model. Results for IBD subtype, diagnosis period, sex, and age group are reported as odds ratios (ORs) with 95% confidence intervals (CIs).

In additional descriptive analyses, we assessed the first prescribed treatment class, the timing of the first locally acting corticosteroid prescription relative to the first prescription of a main treatment (mesalazine, thiopurines, methotrexate, biologics, or targeted synthetic therapies), and the co-prescription of selected treatment classes, defined as prescriptions of both treatment classes within 90 days of each other. For these analyses, biologics were further differentiated into TNF inhibitors, vedolizumab, and IL-12/23 or IL-23 inhibitors. Among UC patients with a biologic prescription, we further assessed mesalazine prescriptions before or on the date of and after the first biologic prescription.

Because multiple statistical comparisons were performed, a two-sided *p*-value of <0.001 was considered statistically significant. All analyses were performed using SAS version 9.4 (SAS Institute, Cary, NC, USA).

## 5. Conclusions

This large real-world study demonstrates substantial differences in prescription patterns between the 2015–2019 and 2020–2024 diagnosis periods among pharmacologically treated patients with newly diagnosed IBD managed in office-based gastroenterology practices in Germany. Biologics were prescribed more frequently in the later diagnosis period, particularly in CD, whereas thiopurine and mesalazine prescriptions were less frequent. Nevertheless, conventional therapies remained an important component of pharmacological management. Prescription patterns differed substantially between CD and UC and across age groups, whereas sex-related differences were limited. Despite the increasing availability of advanced therapies, systemic corticosteroids remained frequently prescribed around the time of diagnosis, highlighting the importance of further investigating corticosteroid exposure, treatment duration, and opportunities for steroid-sparing strategies in routine care.

Overall, these findings provide contemporary real-world evidence on evolving pharmacological prescription patterns in office-based gastroenterology practices in Germany. The continued prescription of mesalazine in a substantial proportion of patients with CD, despite its limited role in current treatment recommendations, warrants further investigation. Future studies incorporating information on disease activity, phenotype, treatment sequencing and duration, cumulative corticosteroid exposure, and clinical outcomes may help clarify the factors underlying treatment selection and determine how evolving treatment strategies translate into routine clinical practice.

## Figures and Tables

**Table 1:** Baseline characteristics of the study population.

Characteristics	CD Patients	UC Patients	
	N (%)	N (%)	*p*-Value ^1^
Total	3810 (100%)	6199 (100%)	
Age (Mean, SD)	40.5 (15.5)	44.3 (15.8)	<0.001
Age 15–30	1244 (32.7)	1445 (23.3)	<0.001
Age 31–45	1123 (29.5)	1910 (30.8)	
Age >45	1443 (37.9)	2844 (45.9)	
Women	2089 (54.8)	3074 (49.6)	<0.001
Men	1721 (45.2)	3125 (50.4)	
Diagnosis in 2015–2019	1580 (41.5)	2662 (42.9)	0.148
Diagnosis in 2020–2024	2230 (58.5)	3537 (57.1)	
Private health insurance	297 (7.8)	589 (9.5)	0.004
Statutory health insurance	3513 (92.2)	5610 (90.5)	
Number of physician visits per year during the follow-up (Median, IQR)	8 (5–11)	6 (3–10)	<0.001

Data are absolute numbers (N) and percentages (%), unless otherwise specified. CD: Crohn’s disease; UC: ulcerative colitis; SD: standard deviation; IQR: interquartile range. ^1^ Wilcoxon rank-sum test for continuous variables and the chi-square test for categorical variables.

**Table 2:** Proportion of patients with prescriptions for predefined treatment classes during the observation period, by IBD subtype and diagnosis period.

Therapies	2015–2019		2020–2024	
	CD	UC	CD	UC
	N (%)	N (%)	N (%)	N (%)
Total	1580 (100%)	2662 (100%)	2230 (100%)	3537 (100%)
Mesalazine	656 (41.5) *	2389 (89.7) *	652 (29.2) *	3109 (87.9) *
Locally acting corticosteroids	769 (48.7) *	753 (28.3) *	964 (43.2) *	1117 (31.6) *
Systemic corticosteroids	521 (33.0) *	695 (26.1) *	644 (28.9)	1032 (29.2)
Thiopurines	351 (22.2) *	291 (10.9) *	299 (13.4) *	267 (7.5) *
Methotrexate	2 (0.1)	2 (0.1)	1 (0.0)	1 (0.0)
Calcineurin inhibitors	1 (0.1)	3 (0.1)	4 (0.2)	12 (0.3)
Biologics	509 (32.2) *	388 (14.6) *	1205 (54.0) *	760 (21.5) *
Targeted synthetic therapies	1 (0.1) *	37 (1.4) *	3 (0.1) *	76 (2.2) *

Data are absolute numbers (N) and percentages (%), unless otherwise specified. CD: Crohn’s disease; UC: ulcerative colitis. * *p* < 0.001. Column percentages may sum to >100% because patients could be prescribed more than one treatment class.

**Table 3:** Proportion of patients with prescriptions for predefined treatment classes during the observation period, by IBD subtype and sex.

Therapies	Women		Men	
	CD	UC	CD	UC
	N (%)	N (%)	N (%)	N (%)
Total	2089 (100%)	3074 (100%)	1721 (100%)	3125 (100%)
Mesalazine	741 (35.5) *	2736 (89.0) *	567 (33.0) *	2762 (88.4) *
Locally acting corticosteroids	984 (47.1) *	984 (32.0) *	749 (43.5) *	886 (28.4) *
Systemic corticosteroids	610 (29.2)	807 (26.3)	555 (32.3)	920 (29.4)
Thiopurines	327 (15.7) *	249 (8.1) *	323 (18.8) *	309 (9.9) *
Methotrexate	2 (0.1)	2 (0.1)	1 (0.1)	1 (0.0)
Calcineurin inhibitors	4 (0.2)	8 (0.3)	1 (0.1)	7 (0.2)
Biologics	934 (44.7) *	535 (17.4) *	780 (45.3) *	613 (19.6) *
Targeted synthetic therapies	2 (0.1) *	45 (1.5) *	2 (0.1) *	68 (2.2) *

Data are absolute numbers (N) and percentages (%), unless otherwise specified. CD: Crohn’s disease; UC: ulcerative colitis. * *p* < 0.001. Column percentages may sum to >100% because patients could be prescribed more than one treatment class.

**Table 4:** Proportion of patients with prescriptions for predefined treatment classes during the observation period, by IBD subtype and age group.

Therapies	Age 15–30		Age 31–45		Age > 45	
	CD	UC	CD	UC	CD	UC
	N (%)	N (%)	N (%)	N (%)	N (%)	N (%)
Total	1244 (100%)	1445 (100%)	1123 (100%)	1910 (100%)	1443 (100%)	2844 (100%)
Mesalazine	372 (29.9) *	1285 (88.9) *	378 (33.7) *	1715 (89.8) *	558 (38.7) *	2498 (87.8) *
Locally acting corticosteroids	540 (43.4) *	497 (34.4) *	511 (45.5) *	590 (30.9) *	682 (47.3) *	783 (27.5) *
Systemic corticosteroids	464 (37.3)	515 (35.6)	334 (29.7)	520 (27.2)	367 (25.4)	692 (24.3)
Thiopurines	240 (19.3) *	174 (12.0) *	202 (18.0) *	196 (10.3) *	208 (14.4) *	188 (6.6) *
Methotrexate	0 (0.0)	1 (0.1)	1 (0.1)	1 (0.1)	2 (0.1)	1 (0.0)
Calcineurin inhibitors	1 (0.1)	5 (0.3)	3 (0.3)	4 (0.2)	1 (0.1)	6 (0.2)
Biologics	701 (56.3) *	379 (26.2) *	483 (43.0) *	341 (17.9) *	530 (36.7) *	428 (15.1) *
Targeted synthetic therapies	4 (0.3) *	29 (2.0) *	0 (0.0) *	44 (2.3) *	0 (0.0) *	40 (1.4) *

Data are absolute numbers (N) and percentages (%), unless otherwise specified. CD: Crohn’s disease; UC: ulcerative colitis. * *p* < 0.001. Column percentages may sum to >100% because patients could be prescribed more than one treatment class.

**Table 5:** Association between IBD subtype, diagnosis period, sex, and age, and the prescription of predefined therapies in gastroenterology practices.

Therapies	Odds Ratio (95% CI)				
	UC vs. CD	2020–2024 vs. 2015–2019	Male vs. Female	Age 31–45 vs. 15–30	Age > 45 vs. 15–30
Mesalazine	15.17 (13.66–16.84) *	0.67 (0.61–0.75) *	0.92 (0.83–1.02)	1.16 (1.01–1.32)	1.20 (1.06–1.36)
Locally acting corticosteroids	0.53 (0.48–0.57) *	1.00 (0.92–1.09)	0.85 (0.78–0.92) *	0.96 (0.86–1.07)	0.89 (0.81–0.99)
Systemic corticosteroids	0.91 (0.84–1.00)	1.01 (0.92–1.10)	1.17 (1.07–1.28) *	0.69 (0.62–0.77) *	0.58 (0.52–0.64) *
Thiopurines	0.49 (0.43–0.55) *	0.59 (0.52–0.67) *	1.25 (1.11–1.42) *	0.88 (0.76–1.02)	0.60 (0.52–0.70) *
Methotrexate	0.59 (0.12–2.95)	0.37 (0.07–2.03)	0.53 (0.10–2.90)	1.76 (0.16–19.56)	1.87 (0.19–18.27)
Calcineurin inhibitors	1.98 (0.72–5.49)	2.94 (0.98–8.80)	0.71 (0.29–1.74)	1.01 (0.34–3.02)	0.73 (0.24–2.18)
Biologics	0.28 (0.26–0.31) *	2.00 (1.81–2.20) *	1.10 (1.00–1.20)	0.60 (0.54–0.68) *	0.48 (0.43–0.53) *
Targeted synthetic therapies	18.14 (6.68–49.25) *	1.56 (1.05–2.30)	1.52 (1.05–2.22)	1.05 (0.67–1.67)	0.64 (0.40–1.02)

CD: Crohn’s disease; UC: ulcerative colitis; CI: confidence interval. * *p* < 0.001.

**Table 6:** Definition of the eight predefined treatment classes with active ingredients and corresponding ATC codes.

Treatment Class	Active Ingredient(s)	ATC Code(s)
5-ASA	Mesalazine	A07EC02
Locally acting corticosteroids	Hydrocortisone, budesonide, beclometasone	A07EA02, A07EA06, A07EA07
Systemic corticosteroids	Methylprednisolone, prednisolone, prednisone, hydrocortisone	H02AB04, H02AB06, H02AB07, H02AB09
Thiopurines	Azathioprine, 6-mercaptopurine	L04AX01, L01BB02
Methotrexate	Methotrexate	L04AX03
Calcineurin inhibitors	Ciclosporin, tacrolimus	L04AD01, L04AD02
Biologics	TNF inhibitors (infliximab, adalimumab, golimumab); Integrin inhibitors (vedolizumab); IL-12/23 inhibitors (ustekinumab); IL-23 inhibitors (mirikizumab, risankizumab, guselkumab)	L04AB02, L04AB04, L04AB06, L04AG05 (formerly L04AA33), L04AC05, L04AC24, L04AC18, L04AC16
Targeted synthetic therapies	JAK inhibitors (tofacitinib and filgotinib, approved for UC only; upadacitinib, approved for both UC and CD); S1P receptor modulators (ozanimod, etrasimod, approved for UC only)	L04AF01, L04AF03, L04AF04, L04AE02 (formerly L04AA38), L04AE05

ATC: Anatomical Therapeutic Chemical; 5-ASA: 5-aminosalicylic acid; TNF: tumor necrosis factor; IL: interleukin; JAK: Janus kinase; S1P: sphingosine-1-phosphate.

## Data Availability

The data presented in this study are available on request from the corresponding author due to ethical restrictions.

## Supplementary Materials

The following supporting information can be downloaded at https://www.mdpi.com/article/10.3390/ph19101590/s1. Table S1: First prescribed treatment class and timing of locally acting corticosteroid prescriptions during the observation period (12 months after the index date), by IBD subtype; Table S2: Continued mesalazine prescription after biologic initiation in patients with UC and co-prescription of selected treatment classes during the observation period (12 months after the index date), by IBD subtype.

## Abbreviations

The following abbreviations are used in this manuscript:

5-ASA	5-aminosalicylic acid
ATC	Anatomical Therapeutic Chemical
CD	Crohn’s disease
CI	confidence interval
DA	Disease Analyzer
ECCO	European Crohn’s and Colitis Organisation
IBD	inflammatory bowel disease
ICD-10	International Classification of Diseases, 10th revision
IL	interleukin
IQR	interquartile range
JAK	Janus kinase
OR	odds ratio
S1P	sphingosine-1-phosphate
SD	standard deviation
TNF	tumor necrosis factor
UC	ulcerative colitis
WHO	World Health Organization
