# Supplementary material for: Changing Prescription Patterns in Pharmacologically Treated Patients with Newly Diagnosed Inflammatory Bowel Disease in German Gastroenterology Practices: A Real-World Study, 2015–2024
Source: Pharmaceuticals (Basel). 2026 Oct 8;19(10):1590. doi: 10.3390/ph19101590 (PMC13708217; doi:10.3390/ph19101590)
Supplement: Supplementary file 1 [file pharmaceuticals-19-01590-s001.zip › pharmaceuticals-4591816-supplementary.pdf]

Supplementary Materials

Table S1. First prescribed treatment class and timing of locally acting corticosteroid prescriptions during the observation period (12 months after the index date), by IBD subtype.

	CD	UC
First prescribed treatment class	N=3,810	N=6,199
Mesalazine	658 (17.3)	3,914 (63.1)
Locally acting corticosteroids	1,073 (28.2)	328 (5.3)
Systemic corticosteroids	465 (12.2)	302 (4.9)
Thiopurines	231 (6.1)	138 (2.2)
Calcineurin inhibitors	1 (0.0)	6 (0.1)
Biologics	914 (24.0)	400 (6.5)
Targeted synthetic therapies	0 (0.0)	27 (0.4)
Two or more treatment classes on the same day	468 (12.3)	1,084 (17.5)
Timing of the first locally acting corticosteroid prescription relative to the first main treatment	N=1,733	N=1,870
No main treatment during the observation period	663 (38.3)	148 (7.9)
Before the first main treatment	481 (27.8)	223 (11.9)
On the same day as the first main treatment	252 (14.5)	534 (28.6)
After the first main treatment	337 (19.4)	965 (51.6)

Data are absolute numbers (N) and percentages (%). Main treatment: mesalazine, thiopurines, methotrexate, biologics, or targeted synthetic therapies. CD: Crohn's disease; UC: ulcerative colitis; IBD: inflammatory bowel disease.

Table S2. Continued mesalazine prescription after biologic initiation in patients with UC and co-prescription of selected treatment classes during the observation period (12 months after the index date), by IBD subtype.

	CD	UC
Mesalazine prescriptions in UC patients with biologic prescriptions		N=1,148
Mesalazine before or on the date of the first biologic prescription	–	600 (52.3)
Mesalazine after the first biologic prescription	–	562 (49.0)
Mesalazine before and after the first biologic prescription (continued use)	–	380 (33.1)
Co-prescription among patients with TNF inhibitor prescriptions	N=1,237	N=672
TNF inhibitor and thiopurine	145 (11.7)	93 (13.8)
Co-prescription among patients with biologic prescriptions	N=1,714	N=1,148
Biologic and mesalazine	242 (14.1)*	702 (61.1)*
Biologic and systemic corticosteroids	494 (28.8)*	519 (45.2)*
Biologic and any corticosteroids	751 (43.8)*	660 (57.5)*
Co-prescription among patients with vedolizumab prescriptions	N=233	N=474
Vedolizumab and systemic corticosteroids	68 (29.2)*	214 (45.1)*
Vedolizumab and any corticosteroids	105 (45.1)*	279 (58.9)*

Data are absolute numbers (N) and percentages (%). Co-prescription was defined as prescriptions of both treatment classes within 90 days of each other. Biologics: TNF inhibitors, vedolizumab, IL-12/23 inhibitors, and IL-23 inhibitors. Any corticosteroids: locally acting or systemic corticosteroids. CD: Crohn's disease; UC: ulcerative colitis; IBD: inflammatory bowel disease; TNF: tumor necrosis factor; IL: interleukin. *$p<0.001$ for the comparison between CD and UC (chi-square test).
